# Following the Worms: Detection of Soil-Transmitted Helminth Eggs on Mothers’ Hands and Household Produce in Rural Kenya

**DOI:** 10.4269/ajtmh.17-0072

**Published:** 2017-08-07

**Authors:** Lauren Steinbaum, Jenna Swarthout, John Mboya, Amy J. Pickering

**Affiliations:** 1 Civil and Environmental Engineering, Stanford University, Stanford, California;; 2 Innovations for Poverty Action, New Haven, Connecticut;; 3 Innovations for Poverty Action, Nairobi, Kenya;; 4 Civil and Environmental Engineering, Tufts University, Medford, Massachusetts

## Abstract

Approximately one-quarter of the world’s population is infected with at least one species of soil-transmitted helminth (STH). The role of produce and hands in STH transmission is not well understood. We collected and processed mother hand rinse and garden-grown produce rinse samples from 116 rural households in Kakamega, Kenya, in an area previously identified to have high STH egg contamination in household soil. *Ascaris* was the only STH species detected; 0.9% of hand rinse, 3.5% of leafy produce, and 1.8% of root produce samples had *Ascaris* eggs. Our results indicate produce and hands can carry *Ascaris* eggs. However, due to the low detected prevalence of eggs on hands and produce, and a high prevalence of cooking the produce items tested, these pathways might have a minor contribution to STH exposure in this setting.

Soil-transmitted helminth (STH) infection is common in tropical, low-income countries.^1^ Infection is spread by the fecal–oral route for *Ascaris lumbricoides* and *Trichuris trichiura.* Both hookworm species, *Ancylostoma duodenale* and *Necator americanus*, infect people transdermally through contact with contaminated soil, but *Ancylostoma duodenale* can also be transmitted via the fecal–oral route.^2^ Past work has assessed the prevalence of STH contamination in community soils, on market produce, and on farmers’ hands, but no studies have examined household produce and mothers’ hands.^3–9^

An important limitation of past work is that STH transmission pathways within the household have not been thoroughly explored. Studies have detected fecal bacteria contamination of the household environment (walls, utensils, toys, hands, water, produce, soil),^10–12^ suggesting more work should be done to evaluate STH contamination along these fecal–oral transmission pathways. Our objective was to measure STH egg contamination of mothers’ hands and household produce in an area where STH infection is endemic and STH egg contamination in soil is common.

We collected hand rinses, produce rinses, and household survey data from 116 rural households in Kakamega, Kenya. We selected households enrolled in the control group of the WASH Benefits study, a randomized controlled trial that assessed the impact of WASH (water, sanitation, and handwashing) and nutrition interventions on child health in Western Kenya.^13^ Our study households were visited by field staff from WASH Benefits 10.5 months prior and were given deworming medicine (albendazole) for the entire compound. Additionally, an ongoing national school-based deworming program had been in effect in the study area since 2012.^14,^^15^ A study evaluating the national school-based deworming program in Kenya found the prevalence of STH infection in schoolchildren in Kakamega was 24.8% in 2014.^14^ The prevalence of *Ascaris* was 24.0%, *Trichuris* was 1.2%, and hookworm was 0.1%.^14^ Also, a study in Kakamega in 2014 found that 19.4% of households had at least one species of STH egg present in soil from the entrance to the house. *Ascaris* eggs were present in 13.4% of households and *Trichuris* eggs were present in 7.5% of households.^16^

Field staff collected two hand rinses (right and left hand) from the mother or primary caregiver of the household. In one household, one hand rinse sample was spilled. Up to two produce items were also collected from each household, with a priority of collecting leafy and root vegetables. We collected 231 hand rinses, 218 produce rinses (including 143 leafy vegetables, 55 root vegetables, and 20 other produce items), and 11 blanks. Household survey questions focused on handwashing, gardening, produce storage, and cooking practices. Survey data were collected electronically using SurveyCTO (Dobility, Inc., Cambridge, MA). The study procedures were approved by the Stanford Institutional Review Board (protocol number 23310) and the Kenya Medical Research Institute Ethical Review Committee (SSC no. 2271). Field staff obtained written consent from all study participants on a prior visit, as well as oral consent on sample collection days.

Field staff collected hand rinse samples using sterile 2,041-mL Whirl-Pak bags (Nasco, Fort Atkinson, WI) filled with 80 mL 1% 7× solution. The respondent’s hand was rinsed thoroughly for 30 seconds. Then, field staff scraped gently under all of respondents’ nails with a nail file, rinsed the nail file in the hand rinse bag, and rinsed her hand again for 15 seconds. The process was repeated for both hands, and the nail file was cleaned with ethanol between samples. Blanks were collected by opening hand rinse bags in the field.

Field staff collected produce samples in sterile 2,721-mL Whirl-Pak bags. Up to two produce samples were collected from each household, with leafy and root produce having priority over other produce (Table 1). We chose the priority list based on the growing season, how common the item is in the area, and whether it is typically grown at home. We collected one item for each root vegetable or other produce type and approximately 50 g of leafy vegetables. Hand rinse and produce samples were collected between morning and early afternoon, stored in a cooler box with ice packs, and sent back to our field laboratory within 6 hours.

**Table 1:** Produce sampling priority list

Leafy vegetables	Root vegetables	Other produce
Sukuma (kale)	Sweet potato	Tomato
Managu (black nightshade)	Cassava	Sugarcane
Amaranthus	Potato	Guava
Pumpkin leaves	Carrot	Mango
Cabbage	Arrowroot	Bell pepper
Cowpea (black-eyed pea) leaves		
Other local greens		

Hand and produce rinse samples were analyzed using the same protocol, with the exception that produce were rinsed with 80 mL of 1% 7X for 30 seconds on arrival in the laboratory. Samples were processed immediately after arriving from the field. Our laboratory protocol was based on an existing method used for detection of STH eggs on hands in high STH transmission areas.^9,17^ We adapted it to have a lower detection limit by using a larger rinse volume, zinc sulfate as a flotation solution instead of sugar, and Sedgwick Rafter cells instead of McMaster slides.

Recovery efficiency of produce rinses was determined by seeding leafy and root produce samples with *Ascaris suum* eggs and performing the produce rinse procedure. *Ascaris suum* eggs were procured from Excelsior Sentinel (Trumansburg, NY) and stored in distilled water at 4°C. Approximately 100 eggs (0.5 mL volume) were counted under a microscope using a 10× objective and added to each produce item. Eggs were transferred using an automatic pipette, placed on produce as small droplets, and allowed to dry before processing. Samples were processed with experimental triplicates according to the protocol described earlier. The recovery efficiency was determined by dividing the final egg count by the initial egg count. The lower detection limit was one egg per sample. The recovery efficiency of the hand rinse method was previously reported to be 95.6%.^17^ The mean recovery efficiency of our produce rinse method was 61.4% (standard deviation = 3.5%) for leafy vegetables (romaine lettuce) and 36.8% (standard deviation = 5.8%) for root vegetables (russet potatoes). None of our blanks were positive for STH eggs.

All respondents had visible dirt on her hands or fingernails. We observed the respondent washing her hands with soap in 3.5% of households (4/116) and washing her hands without soap in 12.1% (14/116) of households. Respondents reported 95.1% of leafy vegetables, 98.2% of root vegetables, and 45% of other produce items (including tomatoes, sugarcane, and guava) were grown in their garden. Although 88.8% of leafy vegetables and 92.7% of root vegetables were harvested immediately before sampling, only 40% of other produce were harvested immediately before sampling. Some produce were grown with animal manure, but none were grown with human waste as fertilizer (Table 2). Produce was not typically washed before collection (Table 2). Produce was also rarely stored on the ground or in a closed container (Table 2). All leafy and root vegetables were typically cooked before eating (Table 2).

**Table 2:** Characteristics of leafy, root, and other produce samples

	Leafy vegetables (*N* = 143) *n* (%)	Root vegetables (*N* = 55) *n* (%)	Other produce (*N* = 20) *n* (%)
Produce is from respondent’s garden	136 (95.1)	54 (98.2)	9 (45)
Produce grown with animal manure	56 (39.2)	12 (21.8)	1 (5)
Produce grown with human waste	0 (0)	0 (0)	0 (0)
Produce was washed before sampling	1 (0.7)	2 (3.6)	0 (0)
Harvested immediately before sampling	127 (88.8)	51 (92.7)	8 (40)
Produce stored on the ground	4 (2.8)	4 (7.3)	4 (20)
Produce stored in a closed container	2 (1.4)	0 (0)	2 (10)
Produce is typically cooked before eating	143 (100)	55 (100)	12 (60)

In our study area, we only found *Ascaris* contamination. The prevalence of *Ascaris* eggs was 0.9% (2/231) in hand rinse samples. Both positive hand samples were from the right hand, and each hand had one STH egg. Neither respondent was observed washing her hands before sampling. The low infection prevalence of *Trichuris* (< 2%) and hookworm (< 1%) reported for our study area could explain why we did not detect these STH. Another potential explanation is STH egg contamination on hands and produce is clustered across time and space and our study was limited in geographic and temporal scope. We found less contamination of hand rinse samples than a similar study performed by Gulliver and others, which found 34% of respondents had STH eggs on their hands with a concentration of up to seven eggs per hand.^9^ The difference could be explained by the fact they collected hand rinse samples from farmers using human waste as fertilizer in an area with an STH infection prevalence of 70%. We also found less contamination of hand rinse samples than a study performed by Cranston and others, which found 16.6% of South African schoolchildren in the study had contaminated hands. *Enterobius vermicularis* was the predominant species identified.^18^

We found 3.0% (6/198) of produce rinse samples had *Ascaris* eggs; 3.5% (5/143) of leafy vegetables, 1.8% (1/55) of root vegetables, and 0% (0/20) of other produce was contaminated. A potential explanation for the low prevalence of contamination is most of our samples were harvested directly from the respondents’ gardens (as opposed to purchased from fresh produce markets) and were not cultivated with human waste as fertilizer. Our results suggest growing leafy and root vegetables in household garden soil does not present a high risk of exposure to STH eggs in this setting (where STH infections have been reduced by deworming, but soil STH contamination is still present). Previous work has demonstrated that produce from food markets was more contaminated with *Ascaris* eggs than produce collected from agricultural fields.^6^ Further research should examine the potential for produce purchased from food markets to serve as a transmission pathway of STH eggs into the home.

In the context of compound-level and school-based deworming, our results imply interventions that seek to eliminate STH infection need to account for ongoing environmental transmission of STH after deworming. Consistent handwashing with soap and washing and cooking produce items postharvest could prevent STH egg ingestion events.^19^ However, alternative interventions to interrupt more prevalent STH environmental exposure pathways may be necessary to significantly reduce STH infections in similar settings.
